# Semi-purified Antimicrobial Proteins from Oyster Hemolymph Inhibit Pneumococcal Infection

**DOI:** 10.1007/s10126-024-10297-w

**Published:** 2024-03-02

**Authors:** Kate Summer, Lei Liu, Qi Guo, Bronwyn Barkla, Kirsten Benkendorff

**Affiliations:** 1 Faculty of Science and Engineering, Southern Cross University, Military Road, Lismore, NSW 2480 Australia; 2 National Marine Science Centre, Southern Cross University, 2 Bay Drive, Coffs Harbour, NSW 2450 Australia

**Keywords:** Antimicrobial resistance, Antimicrobial peptides, Bivalve, Mollusc, Natural products, Drug discovery, Pneumonia

## Abstract

Pneumococcal infections caused by *Streptococcus pneumoniae* are a leading cause of morbidity and mortality globally, particularly among children. The ability of *S. pneumoniae* to form enduring biofilms makes treatment inherently difficult, and options are further limited by emerging antibiotic resistance. The discovery of new antibiotics, particularly those with antibiofilm activity, is therefore increasingly important. Antimicrobial proteins and peptides (AMPs) from marine invertebrates are recognised as promising pharmacological leads. This study determined the in vitro antibacterial activity of hemolymph and unique protein fractions from an Australian oyster (*Saccostrea glomerata*) against multi-drug-resistant *S. pneumoniae*. We developed a successful method for hemolymph extraction and separation into 16 fractions by preparative HPLC. The strongest activity was observed in fraction 7: at 42 µg/mL protein, this fraction was bactericidal to *S. pneumoniae* and inhibited biofilm formation. Proteomic analysis showed that fraction 7 contained relatively high abundance of carbonic anhydrase, cofilin, cystatin B–like, and gelsolin-like proteins, while surrounding fractions, which showed lower or no antibacterial activity, contained these proteins in lower abundance or not at all. This work supports traditional medicinal uses of oysters and contributes to further research and development of novel hemolymph/AMP-based treatments for pneumococcal infections.

**Supplementary Information:**

The online version contains supplementary material available at 10.1007/s10126-024-10297-w.

## Introduction

Pneumococcal infections, caused by the bacterium *Streptococcus pneumoniae*, represent a significant public health burden. Pneumococcal pneumonia, affecting the lower respiratory system, results in over 2.5 million deaths each year, over a third of which are children under 5 years of age (Brooks and Mias 2018; McAllister et al. 2019; WHO 2021). Older people are also highly susceptible to pneumonia, which is a leading cause of hospitalisation and mortality in this demographic (Dadonaite and Roser 2019; Dirmesropian et al. 2019; Tong et al. 2018). Infections of the upper respiratory system caused by *S. pneumoniae* (e.g. tonsilitis, otitis media and sinusitis) are extremely common, representing the most frequent reason for paediatric medical presentations and antibiotic prescriptions (Jin et al. 2021); they are not typically fatal, but symptoms and more serious complications can significantly impair quality of life (Jin et al. 2021). Other clinical presentations of pneumococcal infections include bacterial meningitis and bacteraemia (CDC 2022), while secondary bacterial co-infection by *S. pneumoniae* often leads to increased severity of viral respiratory infections, such as influenza and SARS-CoV-2, contributing to epidemiological data for these diseases (Morris et al. 2017; Rudd et al. 2016; Zhu et al. 2020).

The success and persistence of *S. pneumoniae* are attributed to its ability to form enduring biofilms: populations of bacterial cells embedded in a self-secreted polymeric matrix, which enables surface adhesion and evasion of treatments and host immune defences (Moscoso et al. 2009; Yadav et al. 2020). The high prevalence and impact of pneumococcal infections, their tolerance to treatment in biofilms, and the overuse of antimicrobial agents have contributed to the development of resistant mechanisms (CDC 2019). *S. pneumoniae* is now resistant to one or more relevant antibiotics (e.g. penicillin and derivatives, fluoroquinolones, and macrolides) in 30–50% of infections, limiting empirical treatment options (Appelbaum 2002; Cantón et al. 2002; Cherazard et al. 2017). While pneumococcal conjugate vaccines are generally effective means of prevention, they cannot offer coverage against the many different and regionally variable *S. pneumoniae* serotypes (Du et al. 2021). New antibiotics with novel mechanisms of action (i.e. not merely derivatives of known compounds), and the added ability to attenuate biofilm formation, are therefore needed to address pneumococcal infections.

Oysters and other bivalve molluscs naturally possess strong chemical defences since they are highly exposed to microbes while filter feeding (Defer et al. 2013). Despite high microbial concentrations in seawater and lack of acquired immune systems, oysters and other marine invertebrates rely on humoral immune defence factors, including antimicrobial proteins and peptides (AMPs) (Benkendorff 2010; Coutellec and Caquet 2016; Gianazza et al. 2021; Hooper et al. 2007; Tincu and Taylor 2004). Compared to other marine invertebrates, the immunology of oysters is well understood due to their commercial and environmental importance (Allam and Raftos 2015; Dupont et al. 2020; Ewere et al. 2020; Wang et al. 2018). The antimicrobial activity of oyster hemolymph and constituent AMPs has been extensively researched in the context of improving resistance to marine pathogens responsible for disease outbreaks affecting the aquaculture industry (Anderson and Beaven 2001a, b; Duperthuy et al. 2010; Green et al. 2016; Hubert et al. 1996; Lokmer and Mathias Wegner 2015; Novoa et al. 2016; Raftos et al. 2014; Rosa et al. 2011; Schmitt et al. 2012; Seo et al. 2013a, b). More recently, attention has turned to applications of oyster AMPs to overcome human pathogens (Defer et al. 2013; Gueguen et al. 2009; Guo et al. 2021; Liu et al. 2008; Nam et al. 2015; Seo et al. 2021; Seo et al. 2013a, b), including methicillin-resistant *Staphylococcus aureus* (MRSA) (Defer et al. 2009, 2013; Erdem Büyükkiraz and Kesmen 2021; Hoang and Kim 2013; Loth et al. 2019; Mao et al. 2021; Seo et al. 2021, 2017; Seo et al. 2013a, b; Zhang et al. 2018). AMPs are increasingly targeted as antimicrobial drug candidates (Ageitos et al. 2017; Cheung et al. 2015; Defer et al. 2013; Fredrick and Ravichandran 2012; Jorge et al. 2012; Kang et al. 2019; Romano et al. 2022; Shahrour et al. 2019; Sperstad et al. 2011; Tincu and Taylor 2004; Villa and Gerwick 2010; Zanjani et al. 2018) and are also well suited to combat biofilms, although few studies have included biofilm-related measures (Jorge et al. 2012; Raheem and Straus 2019; Shahrour et al. 2019).

Further, oysters are widely used as traditional medicines and functional foods. In traditional Chinese medicine, various preparations of oysters are recommended specifically for symptoms of respiratory infection and inflammatory conditions (Summer et al. 2020; Zhang et al. 2021). In Australia, oysters played a significant role in the general health of Indigenous people for millennia (Lee and Ride 2018; Reeder-Myers et al. 2022) and a range of nutraceutical products are now available claiming to support immune function (e.g. (Gelatin Australia 2023; Deep Blue Health 2023; Bulk Supplements 2023; Swanson 2023; Unichi 2023; Vitatree 2023). Notwithstanding, the bioactive compounds underpinning medicinal/nutraceutical applications require further validation (Summer et al. 2020). The aim of this study was to determine the *in vitro* antibacterial and antibiofilm activity of hemolymph from the Sydney Rock Oyster (SRO), *Saccostrea glomerata*, against *S. pneumoniae* and identify potentially active constituent proteins for further purification and development. More broadly, it may contribute to recognition of SRO as a functional food for respiratory infection and immunity.

## Materials and Methods

### Hemolymph Extraction

**Fig. 1:**
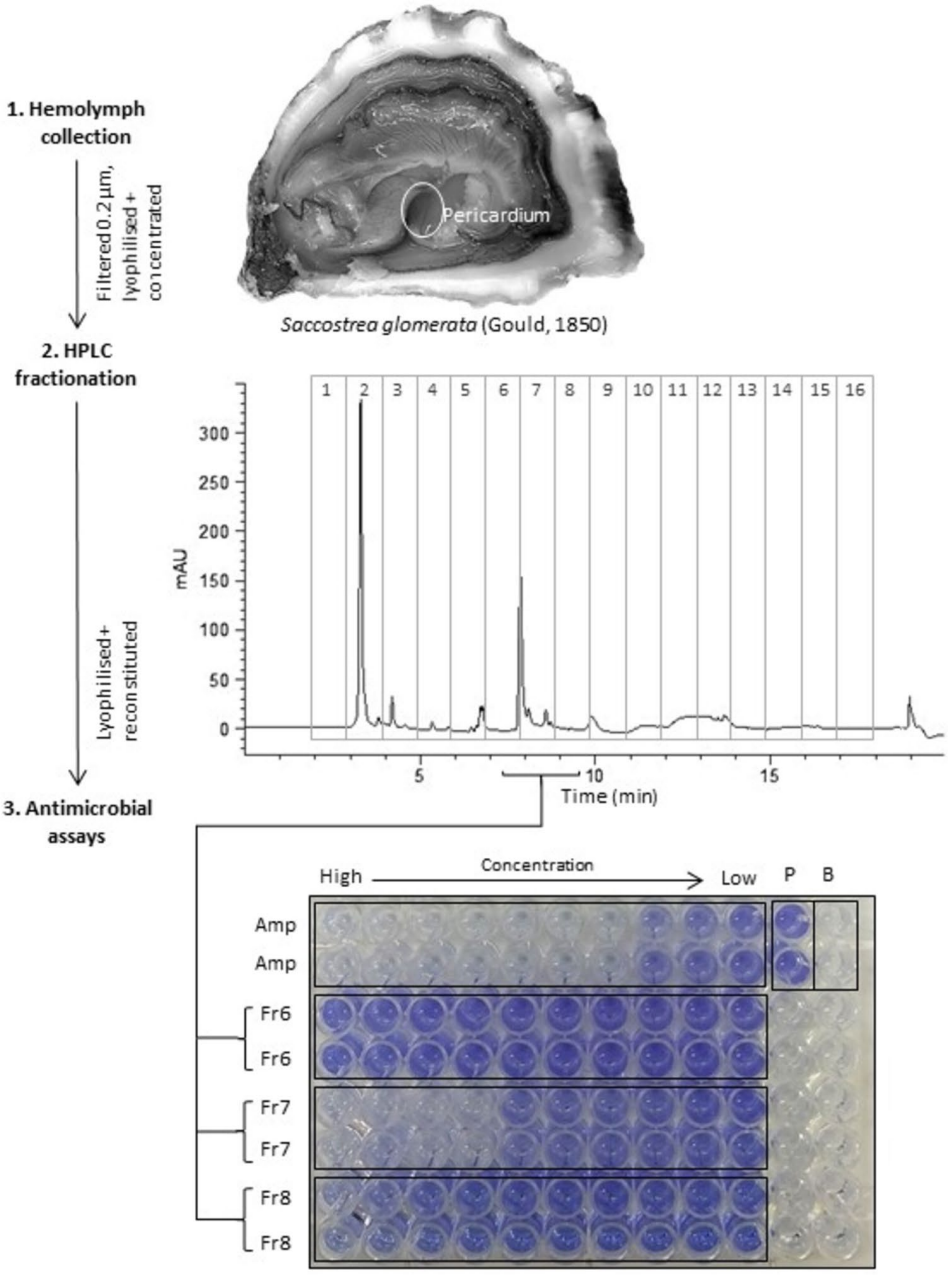
Hemolymph was withdrawn from the pericardial region of *S. glomerata* before fractionation into 16 fractions by preparative HPLC (chromatogram shown at 280 nm, fractions collected at 1-min time slices between 2 and 17 min, described in the ‘Hemolymph Fractionation by Preparative High-Performance Liquid Chromatography (HPLC)’ section). Fractions were tested for antibacterial-biofilm inhibitory activity against *S. pneumoniae*. fractions 6, 7, and 8 showed activity between 100 and 0.2 µg/mL fraction 6 (weak activity), 150 and 0.3 µg/mL fraction 7 (strong activity), and 137 and 0.3 fraction 8 (intermediate activity). All other fractions showed no activity. Ampicillin (Amp) 16–0.03 µg/mL was the positive control. P positive (100%) growth control, B blank. Purple = crystal violet stain reflects density of biofilm-adhered cells

Live SRO were sourced from the Clyde River, Batemans Bay, NSW, Australia, in April 2021 and June 2022 and transported to Southern Cross University, Lismore, NSW, via a commercial supplier. Within minutes of shucking, hemolymph was withdrawn from the pericardial region (Fig. 1) using a sterile syringe and 26 gauge needle (Ewere et al. 2020). Hemolymph was pooled from 40 individuals (resulting in approximately 15 mL per pool) to account for intraspecific variation and increase final protein concentration. Pools were collected in 1.5-mL increments on ice and filtered to remove microbes, hemocytes, and debris by replacing the needle with a 0.2-µm syringe filter and collecting the filtrate in centrifuge tubes (cell-free hemolymph; CFH). Samples were frozen at – 80 °C then freeze-dried over 24 h (Christ Alpha 1–4 LD plus, at – 55 °C and vacuum sealed to 0.035 mbar). Each hemolymph pool resulted in 466 mg (0.03 ± SD) lyophilized powder which was stored at – 80 °C for less than 1 month prior to fractionation.

### Hemolymph Fractionation by Preparative High-Performance Liquid Chromatography (HPLC)

Lyophilized powder from each CFH pool was resolubilized in 2-mL Milli-Q water before fractionation was carried out using an Agilent 1100/1200 HPLC system equipped with a vacuum degasser, quaternary pump, auto-injector, diode array detector (DAD), and fraction collector. The method employed a Phenomenex Jupiter 5u C18, 250 × 10 mm, 300Å column with no temperature control. Absorbance was monitored at 210 and 280 nm using the ChemStation software B.04.03. The mobile phase included 5% acetonitrile (ACN) with 0.05% trifluoroacetic acid (TFA) and Milli-Q water with 0.05% TFA. The elution gradient commenced at 5% ACN, increased to 99% at 15 min, and returned to 5% between 16.5 and 20 min at a flow rate of 5 mL/min. The injection volume was 400 µL with five injections per run (i.e. per hemolymph pool). Sixteen fractions were collected at 1-min time slices between 2 and 18 min, which coincided with peaks observed on the chromatogram (Fig. 1). Fractions were then freeze-dried at − 80 °C (Christ Alpha LOC-1M) over 2 days then vacuum dried for a further 2 days to remove residual solvents, before finally being resolubilized to 300 µL in sterile PBS and aliquoted into Eppendorf tubes, then stored at – 80 °C for use in respective assays.

### Proteomics

#### Protein Quantification

Protein concentration was determined in 96-well plates for crude hemolymph concentrate and each hemolymph fraction according to the procedure described by Bradford (Kruger 2009). Briefly, a series of bovine serum albumin (BSA) standards were prepared ranging from 0.5 to 7.0 μg BSA in 10 μL PBS. Five microlitres of hemolymph and fraction samples was diluted in 5 μL PBS. Then, 100 μL protein dye reagent (Bio-Rad, Australia) was added to each well. Absorbance was measured at OD 595 within 15 min of commencing the assay. Samples and standards were repeated in triplicate in each assay. Absorbances were blank corrected, and sample protein concentrations were estimated according to the BSA standard curve, reported as means ± standard deviation (SD).

#### Protein Separation and Visualization by SDS-PAGE

Proteins comprising each fraction were separated using pre-cast polyacrylamide gels (Bio-Rad Mini-PROTEAN TGX, 10-well). For fractions 6, 7, and 8, a sample representing 1–2 µg total protein was prepared by mixing with 2 × Laemmli sample buffer (Bio-Rad, Australia) in a 1:1 volume ratio; the total sample volume ranged between 6 and 8 µL. Sample volumes were higher (~ 20 µL) for other fractions since protein concentrations were low. Ten microliters of molecular weight marker (Precision Plus Protein™ Dual Xtra pre-stained protein standard, Bio-Rad, Australia) was positioned in the first well of each gel, and 10 µL Laemmli buffer was added to the remaining empty wells. The gels were electrophoresed at 140 V in a buffer solution (25 mM Tris, 192 mM glycine, 0.1% SDS; Bio-Rad, Australia) and run for approximately 40 min until the sample reached completion. Gels were carefully removed from casing and fixed with a solution of 40% ethanol and 10% acetic acid for 30 min, then rinsed with Milli-Q water before staining overnight with QC colloidal Coomassie blue (Bio-Rad, Australia) with gentle agitation. Gels were then de-stained with a solution of 50% (v/v) methanol and 10% (v/v) acetic acid in Milli-Q water with gentle agitation over 3 h. The gel was photographed on a white light illuminating box. Finally, bands were cut using a sterile blade and placed in respective Eppendorf tubes to prepare for analysis.

#### Protein Identification by HPLC–MS/MS

Subsamples of whole hemolymph fractions and gel bands were trypsin digested first at 5 °C for 30 min and then at 37 °C overnight, and the resulting peptides were recovered by three extractions with 35 μL of 50% (v/v) acetonitrile with 2% (v/v) formic acid. The extracts were dried in a vacuum centrifuge and redissolved in 15 μL of 5% formic acid before being analysed by microflow HPLC/MS MS/MS on an Eksigent, Ekspert nanoLC 400 system (SCIEX, Canada) coupled to a triple time-of-flight (TOF) 6600 mass spectrometer (SCIEX, Canada) equipped with a micro Duo IonSpray ion source. A volume of 5 µL from each extract was injected onto a 5 mm × 300 μm, C18, 3-μm trap column (SGE, Australia) for 6 min at 10 μL/min. The trapped tryptic peptide extracts were then washed onto the analytical 300 μm × 150 mm Zorbax 300SB-C18 3.5-μm column (Agilent Technologies, USA) at a flow rate of 3 μL/min and a column temperature of 45 °C. Linear gradients of 2–25% solvent B over 60 min at 3 μL/minute flow rate, followed by a steeper gradient from 25 to 35% solvent B in 13 min, then 35 to 80% solvent B in 2 min, were used for peptide elution. The gradient was then returned to 2% solvent B for equilibration prior to the next sample injection. Solvent A consisted of 0.1% formic acid in Milli-Q water and solvent B contained 0.1% formic acid in ACN. The micro ion spray voltage was set to 5500 V, de-clustering potential (DP) 80 V, curtain gas flow 25, nebulizer gas 1 (GS1) 15, heater gas 2 (GS2) 30, and interface heater at 150 °C. The mass spectrometer acquired 250-ms full-scan TOF-MS data followed by up to 30- and 50-ms full-scan product ion data, with a rolling collision energy, in an information dependent acquisition (IDA) scan mode. Full-scan TOF-MS data was acquired over the mass range *m*/*z* 350–2000 and for product ion ms/ms, *m*/*z* 100–1500. Ions observed in the TOF-MS scan exceeding a threshold of 150 counts and a charge state of + 2 to + 5 were set to trigger the acquisition of product ion, ms/ms spectra of the resultant 30 most intense ions. The data was acquired and processed using Analyst TF 1.7 software (ABSCIEX, Canada).

#### Protein Data Analysis

Protein Pilot 5.0.2 (SCIEX, Canada) was used to search spectra against the UniProt Mollusca database (723, 993 entries, 4 Oct 2022). Scaffold 4.8.6 (Proteome Software, USA) was used to validate MS/MS-based protein identification and quantification, whereby identifications were accepted if they could be established at > 99% probability and contained at least two unique peptides. Normalized spectral abundance factor (NSAF) was used for quantification, as described by Yang et al. (2022). Principal component analysis (PCA) (Chanana et al. 2017) and hierarchical clustering (Ward’s method) (Key 2012) were undertaken in R (4.1.0) (Team 2022) using packages ‘heatmap.plus’ and ‘gplots’ for the heatmap and ‘ggplot2’, ‘FactoMineR’, and ‘factoextra’ for PCA to visualise the abundance of unique proteins in Fractions 6, 7, and 8.

### Antibacterial-Antibiofilm Assays

#### Bacteria Preparation

We used *S. pneumoniae* laboratory strain ATCC 51916, which shows multi-drug resistance including broad-spectrum cephalosporins (https://www.atcc.org/products/51916). Cryopreserved bacteria were revived on horse blood agar (HBA) and grown to log-phase over 20–22 h at 37 °C with 5% CO_2_. To prepare the media, cation-adjusted Mueller Hinton II broth (CAMHB) (BD BBL^™^ powder, Thermo Fisher) was prepared in Milli-Q, and defibrinated horse blood (Edwards Group, Australia) was lysed over five freeze-thaw cycles before addition to CAMHB (5% v/v). Isolated colonies were subsampled and suspended in 1 mL media and grown to log-phase in a shaking incubator at 37 °C with 5% CO_2_ for 3–4 h until blank-corrected absorbance was 0.1–0.2, as measured spectrophotometrically at 600 nm (Bio-Rad iMark^™^ microplate reader), which was equivalent to ~ 10^8^ CFU/mL. Stocks were diluted in media to achieve a working suspension of 10^6^ CFU/mL, finally reduced to 5 × 10^5^ CFU/mL in assays. CFU’s were confirmed by plating dilutions of working suspensions used in each assay.

#### Antibacterial-Biofilm Inhibition Coupled Assays

The liquid growth microdilution method was applied in accordance with standard procedures (Clinical and Laboratory Standards Institute 2018), with some modifications for determination of biofilm inhibition as per Summer et al. (2022). We initially focused on CFH, the salt fraction (fraction 2) and protein fractions 6, 7, 8, 9, 12, and 15 which were of interest based on chromatograms and protein data (Fig. 1). Repeat experiments (*n* = 5) then focused on the most active fraction (7) and those directly either side (6 and 8).

Ninety-six-well plates were prepared as follows: 50-μL media were added to all wells; then, 50 µL of samples was added in duplicate to wells in column 1. Tenfold serial dilutions were made before 50-μL bacterial suspension was added. When reconstituted to between 0.3 and 0.5 mL in PBS then diluted in assays, the highest average protein concentrations of fractions 6, 7, and 8 tested were approximately 100.5 µg/mL (± 5.3 SD), 150.7 µg/mL (± 5.1 SD), and 137.8 µg/mL (± 1.0 SD), respectively. CFH was tested at a top concentration of ~ 200 µg/mL protein. The salt fraction contained no protein, but was tested between 0.04 and 25.0% v/v in media.

All plates included duplicate positive-growth media controls, blank media-only controls, and serial ampicillin dilutions (CAS 7177-48-2, Sigma Aldrich, reconstituted in PBS as per Clinical and Laboratory Standards Institute, 2018) as negative controls. Plates were incubated for 20–22 h at 37 °C with 5% CO_2_ then read spectrophotometrically at OD 600 for determination of antibacterial activity (planktonic growth inhibition). The same plates were evaluated for inhibition of biofilm formation by aspirating planktonic cells and media from the wells and rinsing twice with PBS. Remaining biofilms were sprayed with 80% v/v ethanol and allowed to dry, then stained with 200 µL 0.1% crystal violet. After 20 min, excess stain was discarded and plates were again twice-rinsed with PBS. Stained biofilms were solubilized with 200 µL 30% v/v glacial acetic acid and OD was measured at 570 nm.

Minimum inhibitory concentrations (MIC) were recorded as the minimum concentrations inhibiting growth relative to untreated (media-only) blanks (i.e. treatment absorbance ≤ blank absorbance). To determine minimum bactericidal concentrations (MBC), 20-µL solution was removed from the MIC well, diluted in 180 µL sterile PBS, and spread over HBA to identify presence/absence of growth after overnight incubation. All raw measurements were blank corrected. Absorbance measurements from duplicate treatments on each plate were averaged and data from *n* = 5 replicate experiments were used in the analysis Data are reported as means ± standard deviation (± SD) from the five replicate plates. Biofilm inhibition was calculated as percentages relative to respective positive-growth controls:

$$\%\ \text{inhibition}= 100 - \left(\frac{{\text{treatment}}\;-\;{\text{blank}}}{\text{positive growth control}\;-\;{\text{blank}}}\right)\times 100$$

#### Antibacterial-Biofilm Inhibition Statistical Analysis

Five-parameter log-logistic dose-response models (Gottschalk & Dunn 2005) were fit using Markov chain Monte Carlo (MCMC) methods in NIMBLE 0.13.1 (de Valpine et al. 2017) using R (4.2.1) (Team 2022). We modelled the mean observed response (absorbance, inhibition) in experiment $i\in$ of treatment $j\in ({\text{CFH}},\text{ fraction }6,7,8, \text{ampicillin) to}$ protein or antibiotic concentration (${x}_{k}$) as

$$\begin{array}{cc}{y}_{ijk} & \sim \mathrm{Normal}\left({\mu}_{ijk},{\sigma}_{i}\right)\\{\mu}_{ijk} & = c+\frac{{d}_{ij}\;-\;{c}_{ij}}{{\left(1\;+\;{\left(\frac{{x}_{k}}{{e}_{ij}}\right)}^{{b}_{ij}}\right)}^{{g}_{ij}}}\end{array}$$

where $c$ and $d$ are the highest and lowest responses, respectively; $e$ is the median effective concentration (EC_50_); $b$ is the slope at $e$, and $g$ allows for curve asymmetry (Gottschalk and Dunn 2005). The $\sigma$ parameters reflect the measurement error associated with observation. Posterior distributions of parameters with medians and 95% highest posterior density intervals (HPDI) were summarised.

### Carbonic Anhydrase Activity Validation

Commercial carbonic anhydrase from bovine erythrocytes (BovCA) (lyophilized powder, ≥ 2000 W-A units/mg protein, CAS 9001-03-0, Sigma-Aldrich) was tested against *S. pneumoniae* to validate potential activity of the same enzyme in fraction 7. The top concentration of BovCA tested was 150 mg/mL (w/v), comparable to fraction 7 total protein concentrations. Similarity between BovCA and the carbonic anhydrase identified in SRO hemolymph fraction 7 was compared using Protein BLAST (Basic Local Alignment Search Tool, National Centre for Biotechnology Information, https://blast.ncbi.nlm.nih.gov/Blast.cgi?PROGRAM=blastp&PAGE_TYPE=BlastSearch&LINK_LOC=blasthome).

## Results

### Hemolymph Fractionation by High-Performance Liquid Chromatography (HPLC)

**Fig. 2:**
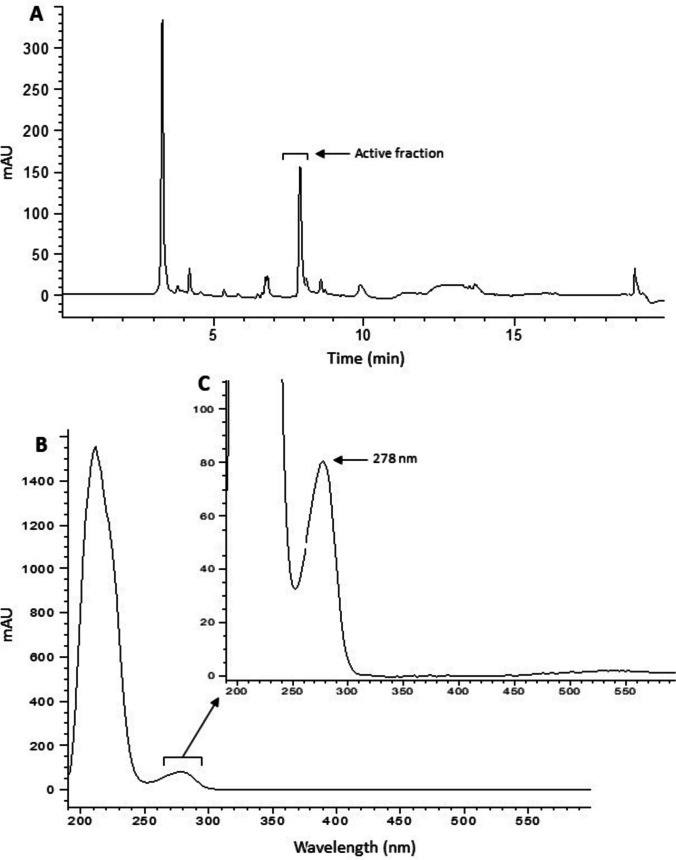
Analysis of a typical concentrated SRO hemolymph sample obtained by preparative HPLC. **A** The chromatogram at 280 nm shows the composition of hemolymph, where fraction 7 showed antibacterial activity (active fraction). **B** Full UV spectra of the active fraction. **C** UV spectra of the active fraction at relevant wavelengths eliminate the presence of other small molecules. Absorbance at 280 nm is specific to proteins (Edelhoch 1967)

Seven major peaks were detected in crude cell-free hemolymph (CFH) analysed by HPLC (Figs. 1 and 2). The average recovery mass of lyophilised powder in each fraction from 2-mL CFH concentrate injections was 2.2 (± 3.1) mg for fraction 6, 12.3 (± 5.7) mg for fraction 7, and 6.9 (± 3.5) mg for fraction 8. In fraction 2, 277.4 (± 121.3) mg salt was recovered. After being resolubilised in phosphate buffered saline (PBS), average protein concentrations estimated by the Bradford assay were 414.6 (± 17.0) mg/mL, 616.0 (± 17.6) mg/mL, 553.3 (± 3.0) mg/mL, and 780.8 (± 22.4) mg/mL for fractions 6, 7, 8, and CFH, respectively.

### Proteomics

SDS-PAGE showed clear differences in the protein profile of each fraction, specifically fractions 6, 7, and 8 (Fig. S1). The bands around 25, 37, 50, and 150 kDa were most intense in fraction 7 (Fig. S1). Proteins identified in each visible band (extracellular superoxide dismutase, SOCS box domain-containing protein, tropomyosin, and carbonic anhydrase) are provided in Supplementary Spreadsheet 2.

**Fig. 3:**
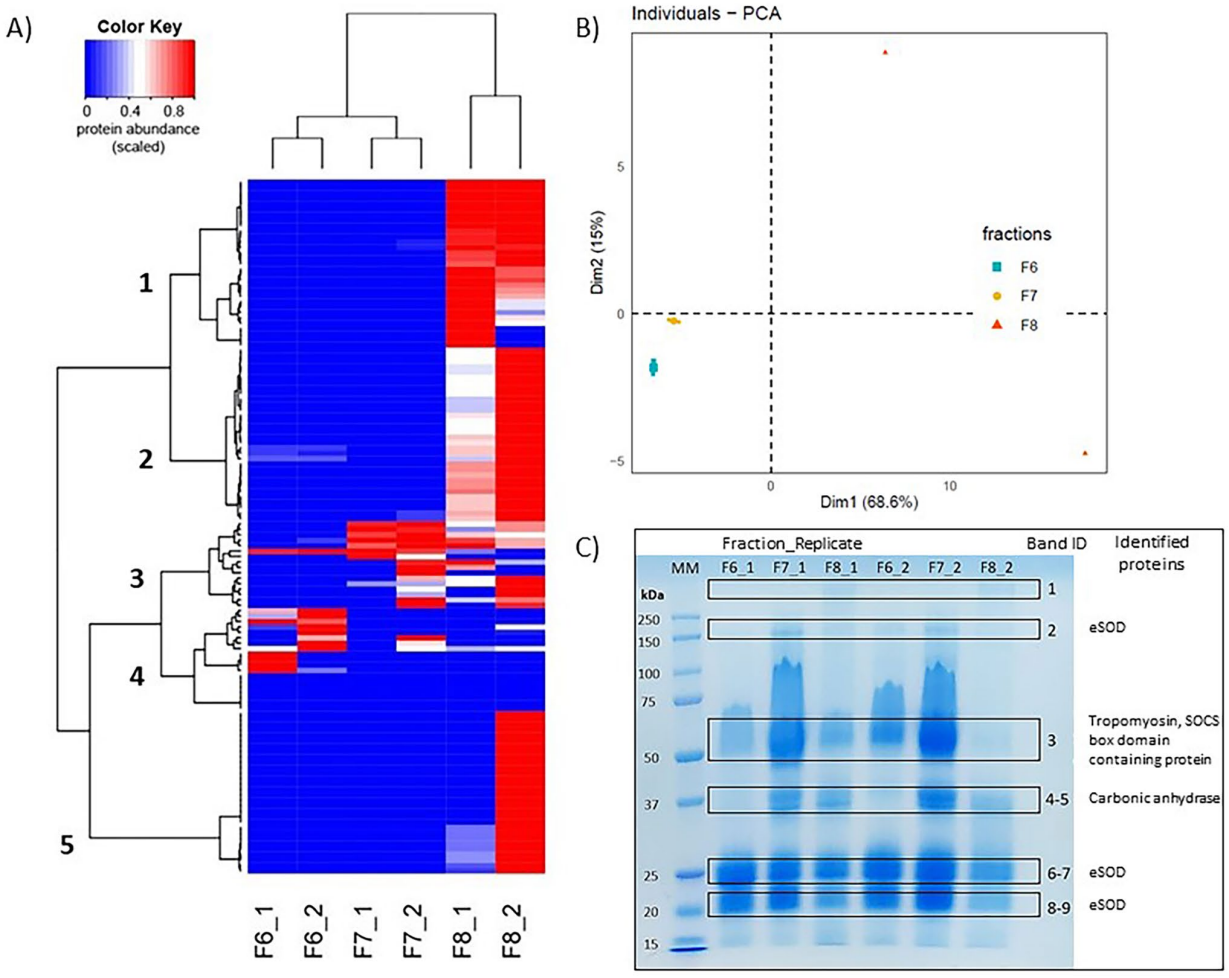
Proteomic analysis of identified proteins in fractions 6, 7, and 8 from two biological replicates of *Saccostrea glomerata* hemolymph. **A** Heatmap showing hierarchical clustering (Ward’s method) of the quantitative values of the identified proteins grouped based on scaled abundance in the respective fractions. **B** PCA of the proteins based on their NSAF. The sample scores for the first and second principal components are plotted. **C** SDS-PAGE gel runs of fractions 6–8 representing 1–2 µg total protein in Laemmli buffer. Detailed protein annotations and complete proteomic data are provided in Supplementary Spreadsheet 1 and Figs. S1 and S2

A total of 128 proteins were identified across fractions 6, 7, and 8, with 16, 17, and 95 proteins in each fraction respectively (Figs. 2 and 3 and Supplementary Spreadsheet 1). Hierarchical clustering of proteomic data indicated five unique clusters (Fig. 3A). Clusters 1, 2, and 5 contained proteins with the highest abundance in fraction 8, while cluster 4 primarily consisted of proteins present in fraction 6. Cluster 3 comprised proteins with a higher abundance in fraction 7 compared to other fractions (Fig. 3A). Proteins in fraction 7 included carbonic anhydrase, tropomyosin, SOCS box domain-containing protein, gelsolin-like protein 2, peptidyl-prolyl cis-trans isomerase, cystatin B–like protein, cofilin, extracellular superoxide dismutase, and heterogeneous nuclear ribonucleoprotein A/B (Supplementary Spreadsheets 1–3; Figs. S1 and S2). Their sequences shared strong similarity (around 99%) at the amino acid level to proteins from *S. glomerata* and other oyster species (*Crassostrea gigas*, *Crassostrea virginica*). PCA was performed on the quantitative values of the proteins identified in these fractions to assess variability among samples (Fig. 3B). The projections of sample scores for the first and second principal components together accounted for 83.6% of the total variance (Fig. 3B). A clear separation was observed for non-overlapping clusters corresponding to different fractions.

### Antibacterial-Biofilm Inhibition Assay

**Fig. 4:**
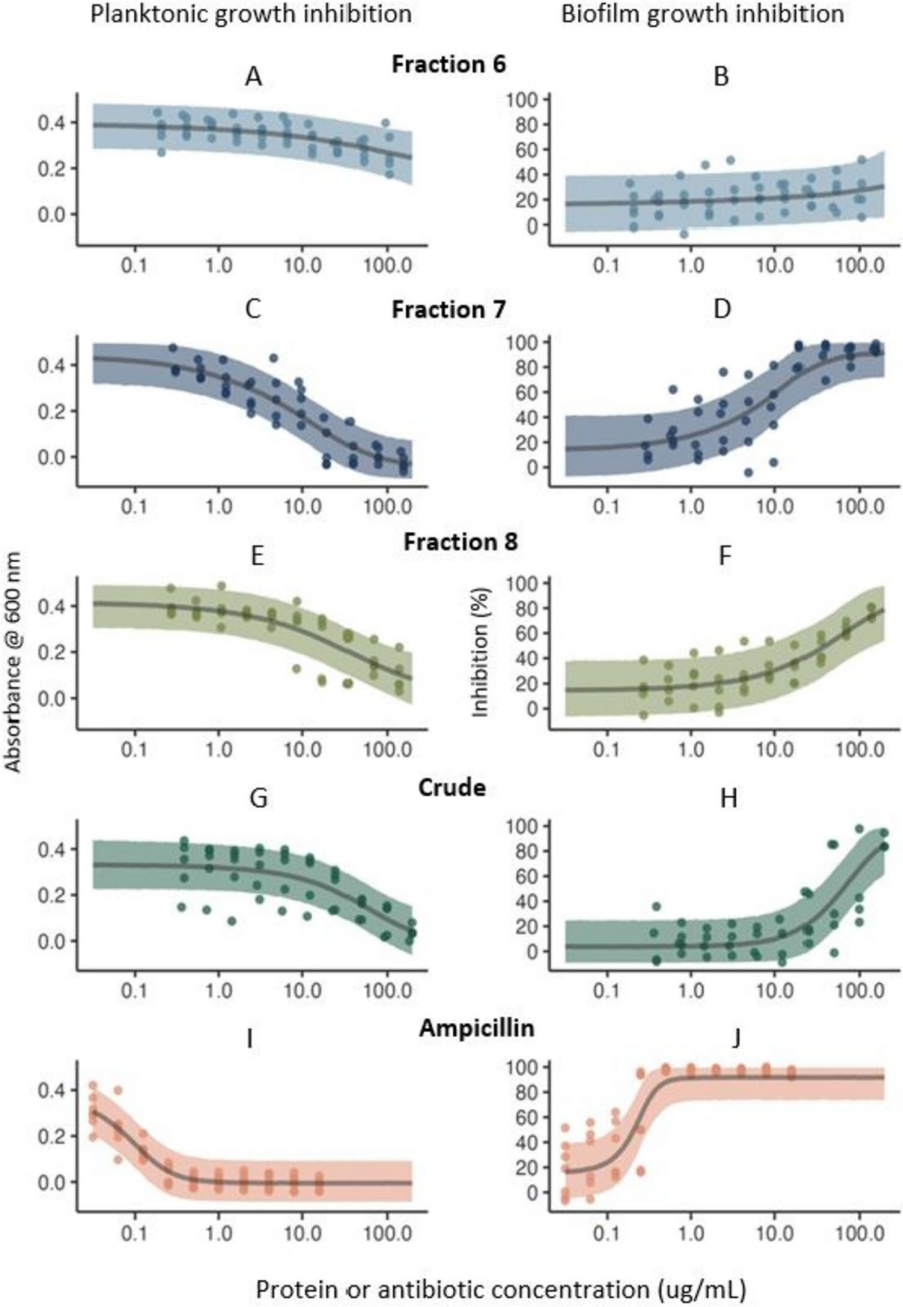
Antibacterial (planktonic growth inhibition) and biofilm-inhibitory activity of HPLC separated SRO hemolymph fractions 6 (**A**, **B**), 7 (**C**, **D**), 8 (**E**, **F**), crude cell-free hemolymph (**G**, **H**), and ampicillin (control, **I**, **J**) against *Streptococcus pneumoniae*. **A**–**J** Observed data (mean duplicate measurements from *n* = 5 replicate experiments) and predictions from the models; left panels = planktonic growth inhibition, i.e. absorbance at 600 nm, measuring the inhibition of planktonic cell growth determined using standard methods; and right panels = inhibition of biofilm formation determined by staining adhered cells, relative to the positive growth control

Minimum inhibitory and bactericidal concentrations (MIC/MBCs) for fraction 6 were not calculable (> 100 µg/mL protein). The average MIC and MBC for fraction 7 were 42 µg/mL protein, while MICs were higher at 138 µg/mL protein for fraction 8 and 141 µg/mL protein for CFH. Fraction 7 was the only treatment, aside from ampicillin, that reliably resulted in 100% inhibition of planktonic growth and biofilm formation (i.e. *c* was equal to 0 absorbance and 100% inhibition, respectively) (Figs. 4 and S3). There was no activity due to treatment with the salt fraction (2), or any other tested fraction (9, 12, 15) against *S. pneumoniae*. The MIC for ampicillin was between 0.06 and 0.25 µg/mL in each assay, in accordance with the CLSI breakpoint (defined standard antimicrobial concentration for quality assurance) of < 0.25 µg/mL for *Streptococcus* sp. Median effective concentrations (EC_50_ values) are summarised in Table 1.

**Table 1:** Protein concentrations (µg/mL) estimated to reduce *S. pneumoniae* growth by 50% (EC_50_) in planktonic (antibacterial) and biofilm (biofilm inhibition) growth forms

**Treatment**	**Antibacterial activity**		**Biofilm inhibition**	
	**EC**_**50**_	95% HPDI	**EC**_**50**_	95% HPDI
Fraction 6	106.36	16.41, 197.99	113.40	15.96, 197.77
Fraction 7	31.37	1.37, 98.99	30.93	0.89, 147.06
Fraction 8	77.81	9.17, 184.33	107.42	26.50, 197.95
CFH	87.73	15.24, 189.93	102.52	30.34, 191.79

*CFH* Cell-free hemolymph

CFH and other selected fractions were also tested against *Pseudomonas aeruginosa* (clinical mucoid strain serotype 2, phagetype 21/44/109/119X/1214 originally isolated from a patient with cystic fibrosis). In these assays, the top concentrations of CFH showed some activity (around 50% inhibition of planktonic growth and 80% inhibition of biofilm formation) but this was attributable to the salt that it contained (i.e. fraction 2) while no other fractions (6, 7, 8, 9, 12, 15) were active against this species (Supplementary Spreadsheets 4 and 5).

## Discussion

AMPs from marine invertebrates exhibit broad structural diversity and bioactivity reflecting the unique evolution of these organisms and have thus garnered significant attention as potential sources of new antimicrobial agents (Barbosa et al. 2020; Bertrand and Munoz-Garay 2019; Cheung et al. 2015; De Zoysa 2013; Defer et al. 2013; Falanga et al. 2016; Hoang and Kim 2013; Hughes and Fenical 2010; Rajanbabu et al. 2015; Semreen et al. 2018; Shukla 2016; Sperstad et al. 2011; Xu et al. 2021; Zhang et al. 2013). Here, we showed that cell-free crude and semi-purified hemolymph from the SRO, *S. glomerata*, had strong activity against *S. pneumoniae*, capable of killing planktonic cells and inhibiting biofilm formation. Effective concentrations of AMPs vary depending on the specific AMP, the degree of purification, test methods, and the target pathogen. Nonetheless, this study and others taken together indicate the value in looking to AMPs and immune components of oysters, as well as other marine invertebrates, for reasons extending beyond their commercial utility, including as sources of new antimicrobial agents.

SRO hemolymph fraction 7 can be considered very effective compared to other tested protein-based mollusc-derived protein extracts and isolated AMPs (Table S1). Antibacterial concentrations of fraction 7 (EC_50_ 31 µg/mL, MIC/MBC 42 µg/mL) are comparable to plasma (hemolymph) protein isolated defensins, proline-rich peptides, and other AMPs from the Pacific oyster, *Crassostrea gigas*, which are considered among the most promising antimicrobial leads (Table S2). The only other study testing molluscan protein extracts against *S. pneumoniae* is by Borquaye et al. (2015); the reported MICs of 1700 and 2000 µg/mL protein derived from crude whole-body extracts of *Galatea paradoxa* (Bivalvia) and *Patella rustica* (Gastropoda) are much higher than the MICs for SRO hemolymph (141 µg/mL) and fraction 7 (42 µg/mL), emphasising the advantage of focusing on hemolymph where antimicrobial factors are most likely to be produced. Notwithstanding their selectivity, the active dose of hemolymph/AMPs reported in this study and others is not as low as conventional antibiotics. However, they are recognised as relatively non-toxic (and so applicable at higher concentrations) and could be particularly useful in combination with conventional antibiotics (Chatupheeraphat et al. 2023; Duong et al. 2021; Zhu et al. 2022); combinations of fraction 7 and conventional antibiotics at sub-MIC concentrations should be investigated.

A process of bioassay-guided sample fractionation by preparative HPLC and protein identification by LC-MS/MS enabled us to narrow down the active components in fraction 7, while retaining their activity (as opposed to using techniques which may have degraded protein structure before testing). The active AMPs in fraction 7 were potentially one or more of the following proteins of relative high abundance with documented antimicrobial activities:

- Gelsolin-like protein 2: gelsolin-derived peptides have been shown to exert direct activity by interacting with bacterial cell membranes (Bucki and Janmey Paul 2006; Bucki et al. 2004; Piktel et al. 2019) while human plasma gelsolin is known to activate other endogenous antimicrobial and anti-inflammatory factors (Weiner et al. 2003).
- Cofilin: is an essential actin regulatory protein, but has been recently recognised as an AMP with lipopolysaccharide binding activity (Li et al. 2022) and identified as part of the proteomic profile of protective mucus on the skin of fish (Honghan et al. 2019; Nigam et al. 2017).
- Cystatin B–like protein: cystatins are family of cysteine protease inhibitors which are ubiquitous in nature and show various antimicrobial and immunomodulatory properties (Shah & Bano 2009); certain classes of cystatins are known to be upregulated in human lung disease (Szpak et al. 2014), while Li et al. found that cystatin-B was present, among a suite of other AMPs, in whole-body extract of the gastropod mollusc *Limax flavus*, which is used as a traditional Chinese medicine for infectious diseases (Li et al. 2020).
- Carbonic anhydrase: showed high abundance in fraction 7. This class of enzymes, which primarily function to catalyse the interconversion of carbon dioxide (CO_2_) and bicarbonate ions (HCO_3_^−^), has been recognised as appealing targets for developing inhibitors or activators with potential antimicrobial applications (Mishra et al. 2020; Flaherty et al. 2021; Supuran 2011; Supuran and Capasso 2020; Supuran 2008). In our experiments, commercial BovCA lacked antibacterial activity so we were not able to validate carbonic anhydrase as the active factor in hemolymph fraction 7. However, it did indicate that the mechanism must not be due to a reduction in environmental carbon dioxide required for *S. pneumoniae* growth. Protein sequence alignment between the BovCA and the oyster carbonic anhydrase showed only 37% similarity, such that the oyster carbonic anhydrase may contain unique regions responsible for activity that are absent from the BovCA (Fig. S4). Further work would be needed to confirm this.

While there is evidence for activity among each of the identified AMPs in fraction 7, the combination may also be important; synergism is a common phenomenon in AMP interaction (Yu et al. 2016). Purity is also important: fraction 8 contained some of the same AMPs (in similar or lower abundance), as well as a diverse range of other proteins which were not present in fraction 7, including some with heterodimerization activity (e.g. histone H4, H2A, H2B) which may have influenced the structure and reduced the activity of active AMP’s present in fraction 8 (Supplementary Spreadsheet 1).

Also, exerting direct bactericidal effects, the AMPs in fraction 7 may also have interfered with the production or activity of adhesins, which normally mediate *S. pneumoniae* attachment and biofilm formation (Izoré et al. 2010; Shivshankar et al. 2009). Concentrations of antibiotics required to overcome biofilms are generally much higher than those required to eliminate planktonic cells (Ciofu et al. 2017). However, in this study, concentrations of fraction 7 required to prevent biofilm formation were not higher than those for antibacterial activity, indicating its usefulness in the biofilm context (Table 1). One of the characteristics that appears linked to both the antimicrobial and antibiofilm efficacies of many AMPs is their dual capacity to act on the environment/extracellular surface, as well as intracellular functions following entry (Duong et al. 2021; Jorge et al. 2012). AMPs with dual antimicrobial and antibiofilm properties have particular relevance to respiratory infections and potential to be used as pre-treatments to medical devices.

Hemolymph and constituent AMPs are generally regarded as having low cytotoxicity. Reported median cytotoxic concentrations (CC_50_ values) for oyster hemolymph tested in cell viability assays range from 750 µg/mL total protein for CFH (Olicard et al. 2005) to 35–88 mg/mL for fractionated hemolymph (Carriel-Gomes et al. 2006). These are substantially higher than the micromolar effective concentrations reported in this study, suggesting it should be possible to optimise a safe and effective dose for targeted antimicrobial applications in future studies. Moreover, the safety of oysters as functional foods/nutraceuticals is demonstrated by their continuous representation in traditional diets and medicine systems, and this study helps to support those applications.

## Conclusions

Here, we present a novel method for the fractionation of SRO hemolymph, which enabled the identification of strong antibacterial-biofilm inhibitory activity in fraction 7 against *S. pneumoniae*. Several proteins are candidates for being the active AMPs, but the combination may also be important. The findings provide a useful basis for further research and development and should be pursued considering that *S. pneumoniae* represents a significant aetiological agent of respiratory infections worldwide and that the discovery of new treatments is a research priority. The findings also support the use of oysters as functional foods and traditional medicines for respiratory infection. Further research into the proteins identified in fraction 7 is needed to evaluate their clinical usefulness. This study contributes to the growing body of research recognising AMPs from marine invertebrates as useful pharmacological leads.

## Supplementary Information

Below is the link to the electronic supplementary material.

Figure S1 SDS-PAGE gel runs of 16 fractions of SRO hemolymph. Figure S2 Hierarchical clustering (Ward’s method) heat map of identified proteins with annotations correlating to data supplied as supplementary in Fractions 6, 7 and 8 of SRO hemolymph. Figure S3 Images of S. pneumoniae cells showing the antimicrobial activity of Fraction 7 (PDF 632 KB)

Supplementary Spreadsheet 1 Identified proteins in Fractions 6, 7 and 8. Supplementary Spreadsheet 2 Identified proteins in SDS-PAGE bands. Supplementary Spreadsheet 3 Review of proteins in Fraction 7. Supplementary Spreadsheet 4-5 Antimicrobial data (XLSX 234 KB)

Supplementary file3 (DOCX 64 KB)

## Data Availability

No datasets were generated or analysed during the current study.
