# Supplementary material for: Semi-purified Antimicrobial Proteins from Oyster Hemolymph Inhibit Pneumococcal Infection
Source: Mar Biotechnol (NY). 2024 Mar 2;26(5):862–75. doi: 10.1007/s10126-024-10297-w (PMC11480171; doi:10.1007/s10126-024-10297-w)
Supplement: Supplementary file 1 — Figure S1 SDS-PAGE gel runs of 16 fractions of SRO hemolymph. Figure S2 Hierarchical clustering (Ward’s method) heat map of identified proteins with annotations correlating to data supplied as supplementary in Fractions 6, 7 and 8 of SRO hemolymph. Figure S3 Images of S. pneumoniae cells showing the antimicrobial activity of Fraction 7 (PDF 632 KB) [file 10126_2024_10297_MOESM1_ESM.pdf]

Semi-purified antimicrobial proteins from oyster hemolymph inhibit pneumococcal infection

Kate Summer, Lei Liu, Qi Guo, Bronwyn Barkla, and Kirsten Benkendorff

SUPPLEMENTARY FIGURES

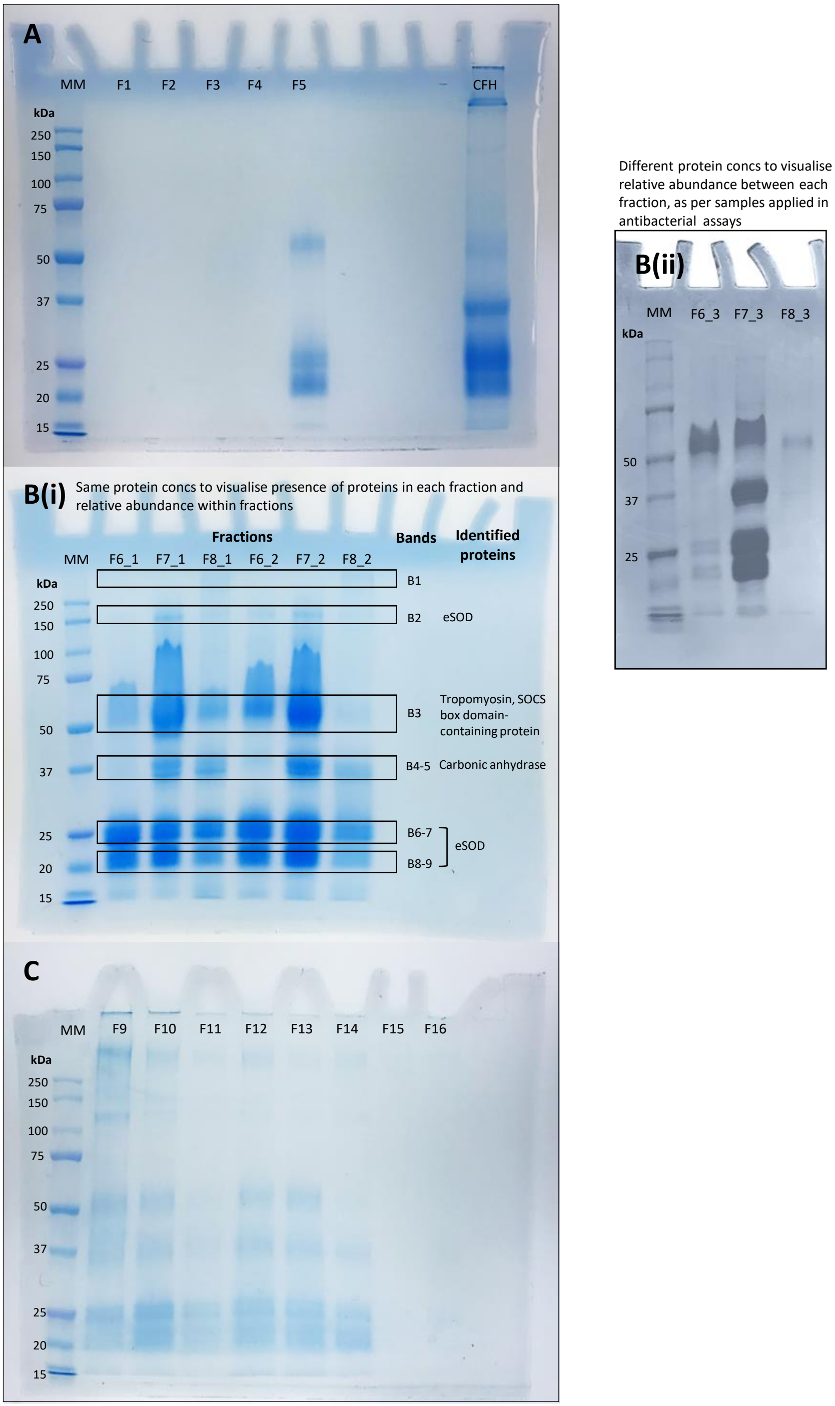

Figure S1: SDS-PAGE gel runs of 16 fractions of SRO hemolymph. Figure Bi= Fraction 6, 7 and 8 from two biological replicates of 6-8 µL representing 1-2 µg total protein in Laemmli buffer. CFH= cell-free hemolymph. Figures A and C= one biological replicate of ~20 µL representing much lower protein concentrations. Figure Bii inset= Fraction 6, 7 and 8 showing relative abundance when all samples added 10 µL at 25% concentration in buffer. Molecular marker (MM) was positioned in the first well of each gel and buffer was added to empty wells. Proteins in each band were identified by HPLC-MS/MS; proteins with high relative abundance in Fraction 7 were likely responsible antimicrobial for activity. Multiple bands corresponded to the same protein for various reasons (protein isoforms, degradation, complexes, etc.). Some proteins (e.g. cofilin, gelsolin-like protein 2, cystatin) were not visible or detected due to having lower abundance than dominant proteins (e.g. carbonic anhydrase, extracellular superoxide dismutase [eSOD], tropomyosin) but may still contribute to overall activity.

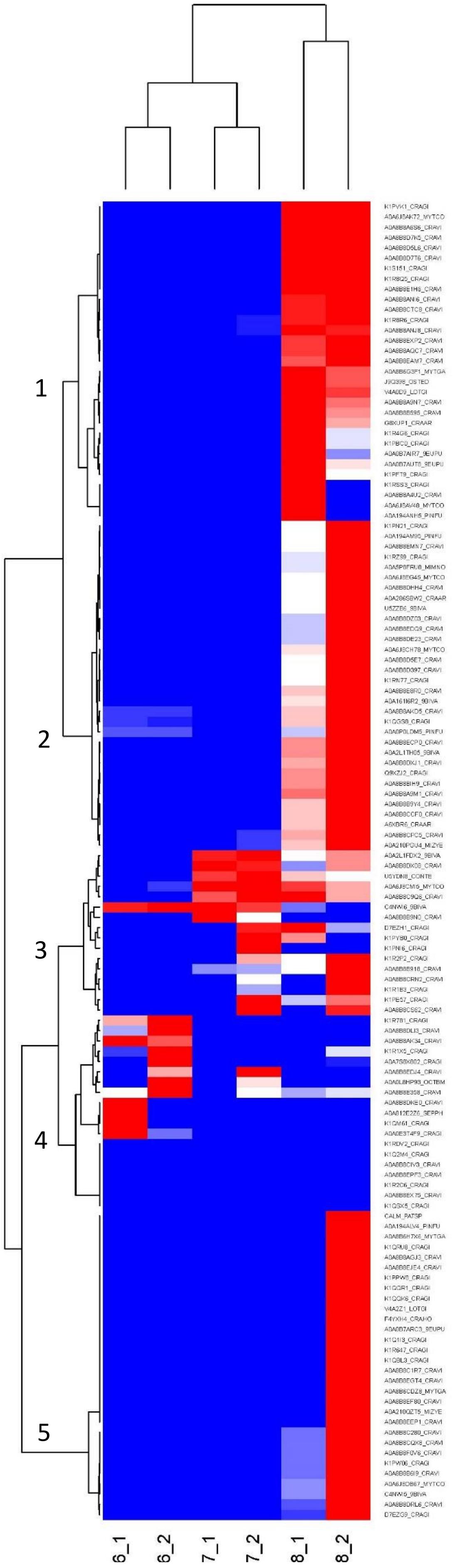

Figure S2: Hierarchical clustering (Ward's method) heat map of identified proteins with annotations correlating to data supplied as supplementary in Fractions 6, 7 and 8 from two biological replicates of hemolymph from the Sydney Rock Oyster, *Saccostrea glomerata* with numbered groupings based on scaled abundance in respective fractions.

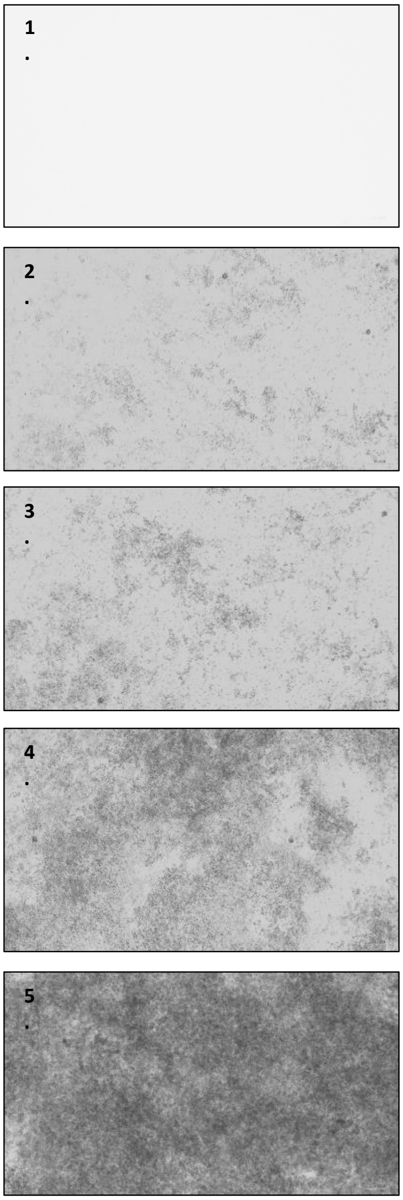

Figure S3: Images of *S. pneumoniae* cells showing the antimicrobial activity of Fraction 7 at 1) 150 µg/mL, 2) 75 µg/mL, 3) 37 µg/mL, 4) 15 µg/mL protein, compared to 5) the positive growth control, taken at 20x magnification using a Nikon Eclipse Ts2 inverted microscope.

```
BovCA  3     SPDWGYDGENGPEHWGKLYPIANGNNQSPIDI-KTSETKRDPSLKPLSVS-YNPATAKEI  60
            + DW Y GE+G   W   YP      QSPID  KT +   D  L     + ++  +   +
SROCA  16   AADWHYTGEHGTSDWPHEYPDCGLVRQSPIDFPKTEDMTYDSKLTQFQFTGFDDLSRYSL  75

BovCA  61   V--NVGHSFHVNFEDSDNRSVLKGGPLSESYRLRQFHFHWGITDDCGSEHLVDGAKFSAE  118
            V  N GH+  +     D   +++GG L   ++  QFHFHWG +D+ GSEH  DG  F  E
SROCA  76   VLHNNGHTAVIKVTGGD--LLVEGGGLPGRFKTAQFHFHWGHSDNEGSEHTFDGHSFPLE  133

BovCA  119  LHLVHWNSAKYPSFADAASQ-ADGLALIGVLVKVGQAN-PNLQKVLDALKAVKNKNKKAP  176
            LH+V++N  KY S ADAAS+  DGLA++G   +V   N  ++  +++ L  V  K    P
SROCA  134  LHIVNYNE-KYGSLADAASKDLDGLAVLGFWYEVSHNNNDDIAPLIEQLSHVPTKGSSVP  192

BovCA  177  FTNFDPSVLLP-----PSLDYWAYSGSLTHPPLHESVTWIIFKETISVSSEQLAQFRSLL  231
             T F+ + LLP         ++ Y GSLT PP  +SV W +F++TI +SS+QL+ FR+L
SROCA  193  LTGFNLAQLLPIHNIQSKSHFFRYPGSLTTPPCFQSVVWTMFQQTIPISSQQLSMFRALH  252

BovCA  232  ANAEGDREVHIKQNNRPPQPLNGRTVKASF  261
             + E   + ++  N RP QPLNGR +  +F
SROCA  253  EDQELQSDHYLVDNFRPIQPLNGRVIYRNF  282
```

Figure S4: BLAST alignments of protein sequences for commercial carbonic anhydrase from bovine erythrocytes (BovCA) and SRO hemolymph carbonic anhydrase (SROCA). Identities: 101/270 (37%), Positives: 148/270 (54%), Gaps: 14/270 (5%).
