# Supplementary material for: Semi-purified Antimicrobial Proteins from Oyster Hemolymph Inhibit Pneumococcal Infection
Source: Mar Biotechnol (NY). 2024 Mar 2;26(5):862–75. doi: 10.1007/s10126-024-10297-w (PMC11480171; doi:10.1007/s10126-024-10297-w)
Supplement: Supplementary file 3 — Supplementary file3 (DOCX 64 KB) [file 10126_2024_10297_MOESM3_ESM.docx]

**Semi-purified antimicrobial proteins from oyster hemolymph inhibit pneumococcal infection**

Kate Summer, Lei Liu, Qi Guo, Bronwyn Barkla, and Kirsten Benkendorff

SUPPLEMENTARY TABLES

**Supplementary Table 1.** Effective concentrations of molluscan hemolymph, antimicrobial proteins and peptides (AMPs), and crude protein extracts targeting bacteria, viruses and fungi reported in the literature and in this study. Concentrations reported in comparable units to this study are in bold.

| **Source organism/s** | | **Specific protein/peptide extract or compound** | **Target microorganism (H: human, T: terrestrial, or M: marine-associated)** | **Effective protein concentrations** | **Reference** |
| --- | --- | --- | --- | --- | --- |
| **Class**  Family | Species |  |  |  |  |
| **Bivalvia** |  |  |  |  |  |
| Ostreidae  (oysters) | *Saccostrea glomerata* | Crude hemolymph and isolated fractions (carbonic anhydrase) | *Streptococcus pneumoniae, P. aeruginosa* (H) | Fraction 7: MIC **42 μg/mL**, EC50 **31 μg/mL; c**rude hemolymph: MIC 141 ug/mL, EC_50_ 88 μg/mL against *S. pneumoniae*; ineffective against *P. aeruginosa* | This study |
|  | *Crassostrea gigas* | Defensin (Cg-Def) and Cg-BPI bactericidal permeability increasing protein (Cg-BPI) | *Vibrio splendidus* (M) | >10 uM | [40] |
|  |  | Recombinant proline-rich peptide (CgPrp) and CgDef | *Staphylococcus aureus*, methicillin-resistant *S. aureus* (MRSA), *Listeria monocytogenes*, *Bacillus cereus* (H) | MICs **5 to 75 µg/mL** | [58] |
|  |  | Synthesised CgPrp and Cg-Def | *Micrococcus lysodeikticus, Brevibacterium stationi, Microbacterium maritypicum, Escherichia coli, Enterobacter cloacae,* *Erwinia carotovora*, *Klebsiella pneumoniae* (H) | MICs 0.005 to > 20 μM (G+) but no or limited activities against G- and fungi | [51, 100] |
|  |  | Recombinant β-thymosin | *E. coli, Bacillus subtilis,* *Candida albicans* (H) | Significant growth suppression **1 to 50 µg/uL** | [53] |
|  |  | Recombinant C-type lectins, CgCLec-4 and CgCLec-5 | *E. coli*, *S. aureus*, *Pseudomonas aeruginosa* (H)*, Yarrowia lipolytica*, *Vibrio alginolyticus*, *Vibrio vulnificus* (M) | Significant growth suppression **2500 µg/mL** | [101] |
|  |  | Hemolymph arginine kinase (CgAK) | *E. coli* (H) | 0.5 mM significantly decreased CFU counts | [102] |
|  |  | CgPep33 obtained by enzyme digestion | *E. coli, P. aeruginosa, B. subtilis, S. aureus* (H), *Botrytis cinerea,* *Penicillium expansum* (T) | EC_50_ values **18.6 to 48.2 μg/mL** | [55] |
|  |  | BigDef1 (Cg-BigDef1) and separate domains. | Large range of marine and human pathogenic bacteria sp. including clinical isolates of MRSA and *P. aeruginosa* (H) | MICs 0.15 to >10 mM | [103] |
|  |  | CgUbiquitin from gill extract | *Streptococcus iniae* (H), *V. parahemolyticus* (M) | MICs **7.8 to 9.8 μg/mL** | [43] |
|  |  | CgMolluscidin from gill extract | *B. subtilis, M. luteus, S. aureus* (H) | MICs **0.4 to 31.3 μg/mL** | [54] |
|  |  | Recombinant CgPrp and CgDef | *S. aureus,* MRSA*, Listeria monocytogenes, B. cereus* (H) | MICs **0.005 to 0.025 µg/mL** | [104] |
|  | *C. gigas*^1^ | Recombinant defensin MgDefdg | *A. hydrophila, S. aureus* (H) | **50 µg/mL** resulted in 70 to 99% antibacterial activity | [63] |
|  | *C. hongkongensis* | URP20 (upregulated peptide) from hemolymph | *V. alginolyticus*, *V. parahaemolyticus* (M), *E. coli, S. aureus*, *C. albicans* (H) | MICs 1 to 10 μM and MBCs 5 to 20 μM | [62] |
|  | *C. virginica* | Recombinant histone H4 | *V. anguillarum* (M) | **1000 µg/mL** protein causing 82.8% inhibition | [105] |
|  | *C. virginica* | Defensin from gill extract | *Lactococcus lactis, S. aureus, E. coli* (H), *V. parahemolyticus* (M) | MICs **2.4 to 15 µg/mL** | [106] |
| Margaritidae (pearl oysters) | *Pinctada fucata* | Recombinant theromacin protein, from whole body tissue | *E. coli, S. aureus* (H), *V. parahaemolyticus, B. licheniformis* (M) | The peptides did not show significant antimicrobial activity, except Cg-Prp22–36 which was weakly antibacterial at 100 μM against *M. lysodeikticus* | [52] |
|  | *P. fucata* | Structurally altered histone-derived AMP | *A. hydrophila, V. parahaemolyticus* (M), *B. subtilis, E. coli, M. luteus, S. aureus, P. aeruginosa* (H) | Significant reduction in growth at **200 ug/mL** | [107] |
| Mytilidae  (mussels) | *M. edulis* | Antimicrobial peptides from hemolymph | *Alteromonas carrageenovora*, *Pseudomonas alginovora, Cytophaga drobachiensis, Neurospora crassa, Fusarium culmorum* (T) | MIC range from 0.3 to 40 μM | [108] |
|  | *Mytilus galloprovincialis* | Myticin C and hemolymph | Ostreid herpesvirus 1 (OsHV-1) (M) and human herpes simplex viruses 1 (HSV-1) and 2 (HSV-2) (H) | Significant reduction in OsHV-1 load, not cytotoxic at **200 µg/mL**, not protective against HSV-1/2 infection | [109] |
|  | *M. galloprovincialis* | Peptides extracted from hemocytes, Myticin A and B | *M. luteus, Bacillus megaterium, S. aureus, L. monocytogenes,* *P. aeruginosa, Brucella suis,* *Fusarium oxysporum* (H) | MBCs 1-210 μM | [110] |
|  | *M. galloprovincialis* | Myticin C and 9 peptide fragments | *P. aeruginosa, S. aureus, M. lysodeikticus* (H) | MICs >64 μM for *P. aeruginosa*; MIC 32 μM of 3 peptide fragments for *S. aureus* | [111] |
| Cardiidae, Veneridae, Ostreidae | *Cerastoderma edule, Ruditapes philippinarum, Ostrea edulis* | Acidic extracts of body tissue (40 and 80% solid-phase extraction) | *M. luteus, E. coli, B. megaterium, Y. ruckeri, L. anguillarum,* three *Aeromonas* sp., two *Vagococcus* sp. (H) | MICs **43 to 2560 µg/mL** | [61] |
|  | *C. rhizophorae,* *C. gigas* | Cellular (c) and acellular (a) hemolymph fractions | Human adenovirus (respiratory strain AdV-5) (H) | CC_50_ 1900–3600 μg/mL; EC_50_ **500-1600 µg/mL** | [112] |
| Donacidae, Patellidae^2^ | *Galatea paradoxa,* *Patella rustica* | Crude peptide extract of body | *S. aureus, S. pneumoniae, K. pneumoniae, P. aeruginosa* (H) | MICs **1700 to 2000 µg/mL** | [98] |
| Veneridae, Donacidae | *Pitar erycina, Latona cuneata^3^* | Crude proteins from flesh acidic extract | *B. subtilis, P. aeruginosa, Salmonella typhi, Shigella flexneri, E. coli* (H) | **1230 ug/mL** (ZI) | [113] |
| **Gastropoda** |  |  |  |  |  |
| Ampullariidae | *Pomacea poeyana* | Peptides from whole body extract | *P. aeruginosa, L. monocytogenes, K. pneumoniae* (H) | **30 to 100 ug/mL** (ZI) | [114] |
| Calyptraeidae, Buccinidae | *Crepidula fornicata, Buccinum undatum* | Acidic extracts of body tissue (40 and 80% solid-phase extraction) | *M. luteus, E. coli, B. megaterium, Y. ruckeri, L. anguillarum,* three *Aeromonas* sp., two *Vagococcus* sp. (H) | MICs **130 to 2400 µg/mL** | [61] |
| Haliotidae | *Haliotis tuberculata* | Haliotisin polypeptides | *B. subtilis* (H), *Erwinia carotovora* (P) | MICs 0.5 to 5 μM | [115] |
| Muricidae | *Rapana venosa* | Hemolymph peptides | *S. aureus, K. pneumoniae* (H) | Up to 80% growth inhibition depending on the fraction, **113–598 μg/mL** | [116] |
|  | *R. venosa* | Hemocyanin (RvH), glycosylated (RvH-c) and non-glycosylated (RvH-b) subunits | Respiratory synctial virus (H) | RvH-c 1000 μg/mL | [117] |
| Muricidae, Helicidae | *R. venosa,* *Helix aspersa* | Hemocyanins and structural subunits | *S. aureus, E. faecium, S. epidermidis, S. pyogenes, E. coli, P. aeruginosa* (H) | MIC: 6.5 μM βc-HaH; MIC not calculated for RvH1 (1.25–10 μM) | [118] |
| Olividae | *Agaronia hiatula*^4^ | Acid-acetone peptide extract of body | *S. aureus, P. aeruginosa, K. pneumoniae* (H) | MIC (**µg/mL**): **2500** *S. aureus*, **390** *P. aeruginosa*, **1250** *K. pneumoniae*; MBC (**µg/mL**): **2500** *S. aureus*, **1250***P. aeruginosa*, **>2500** *K. pneumoniae* | [119, 120] |
| Onchidiidae | *Peronia peronii* | Dolabellanin B2 (AMP) | *S. aureus, P. aeruginosa, K. pneumoniae* (H) | MICs: **10–25 µg/mL** | [121] |
| Solenidae | *Solen grandis* | 2 sialic acid-binding lectin recombinant proteins (rSgSABL-1, -2) | *S. aureus, M. luteus* (H) | **100 μg/mL** (phagocytosis), **90 μg/mL** (microbe agglutination, and encapsulation) | [122] |
| **Cephalopoda** |  |  |  |  |  |
| Octopodidae | *Octopus vulgaris* | Peptide (OctoPartenopin) (crude + 6 HPLC fractions + 5 synthetized fractions) from suckers | *S. aureus, P. aeruginosa* (H) | MIC_80_ (**μg/mL**) **50–200** *S. aureus*, **50- >300***P. aeruginosa*; 80 μM peptides inhibit and eradicate up to 60% biofilm formation | [123] |

Abbreviations: MIC: minimum inhibitory concentration, MBC: minimum bactericidal concentration, G+: Gram-positive, G-: Gram-negative, ZI: causing zones of inhibition on agar

Taxonomy checked against the World Registry of Marine Species (https://www.marinespecies.org/):

^1^ *Magallana gigas* in original article

^2^ Gastropoda

^3^ *Donax cuneatus* in original article

^4^ *Olivancillaria hiatula* in original article
